# Concurrent squamous cell carcinoma and non-hodgkin lymphoma: a rare case report and multidisciplinary approach

**DOI:** 10.1080/23320885.2024.2381757

**Published:** 2024-07-22

**Authors:** Abdulwahid Salih, Ari Abdullh, Hiwa Baba, Aras Qaradaqhy, Rebaz Ali, Rebaz Mohammed, Aso Muhialdeen, Hardi Dhahir, Yadgar Saeed, Fahmi Kakamand

**Affiliations:** a Smart Health Tower, Sulaymaniyah, Iraq; b College of Medicine, University of Sulaimani, Sulaymaniyah, Iraq; c Department of Pathology, Sulaymaniyah Teaching Hospital, Sulaymaniyah, Iraq; d Kscien Organization, Azadi Mall, Sulaymaniyah, Iraq; e Department of Radiology, Shorsh Teaching Hospital, Sulaymaniyah, Iraq; f Hiwa Hospital, Sulaymaniyah, Iraq; g Halabja Emergency Teaching Hospital, Halabja, Iraq

**Keywords:** Skin malignancy, squamous cell carcinoma, surgical resection, non-Hodgkin lymphoma

## Abstract

**Introduction:**

Squamous cell carcinoma (SCC) is a skin malignancy typically treated with surgical resection. Non-Hodgkin lymphoma (NHL) is a group of lymphoid tissue malignancies treated with various strategies, including chemotherapy and radiotherapy.

**Case Presentation:**

A 64-year-old male with prior SCC presented with a new scalp lesion. Examination revealed an elevated, irregular, non-tender lesion with mild yellow discoloration. Imaging showed a scalp lesion, cervical lymphadenopathies, and a well-defined mass. Ultrasonography uncovered lymph node involvement and splenomegaly. Fine needle aspiration, biopsy, and immune stains of the lymph node confirmed NHL. Wide local excision of the scalp lesion, reconstruction, and lymph node biopsies were performed, confirming SCC and NHL. The patient received radiotherapy and chemotherapy.

**Conclusion:**

This unique rare case emphasizes the complex interplay of SCC and NHL, necessitating vigilant SCC patient follow-up.

## Introduction

Squamous cell carcinoma (SCC) is the second most common malignant skin tumor after basal cell carcinoma, accounting for approximately 20% of non-melanoma skin cancers. It predominantly arises on sun-exposed areas, such as the scalp, hands, ears, and lips [1,2]. The primary treatment for SCC involves surgical resection of the tumor with free margins to ensure complete removal [1]. Non-Hodgkin lymphoma (NHL) represents a group of malignant neoplasms originating from lymphoid tissues, primarily affecting lymph nodes. The treatment strategy for NHL is multifaceted, depending on factors such as the disease stage, type, grade, the patient’s age, and overall health. Treatment modalities include chemotherapy, radiotherapy, immunotherapy, or a combination thereof [2,3].

SCC and NHL are two distinct malignancies that typically follow separate clinical trajectories. However, the co-occurrence of SCC and NHL within a single patient is a rare phenomenon [4]. Genuine literature has extensively documented various malignancies occurring independently, but the simultaneous presentation of SCC and NHL remains a rare phenomenon, sparingly reported in scientific literature [4–6].

The incidence of skin cancers and non-Hodgkin lymphoma has increased worldwide, suggesting that these malignancies may share a common etiology [7]. In this manuscript, we present a compelling rare case report of a patient who concurrently diagnosed with SCC and NHL, aiming to provide a comprehensive analysis of this remarkable occurrence, exploring the interplay of factors that may underlie the development of these two distinct malignancies in the same individual.

Additionally, we aimed to elucidate the diagnostic challenges, treatment complexities, and clinical implications associated with synchronous SCC and NHL. By examining this case, we hope to expand our understanding of the intricate relationships between cancer subtypes and contribute to the broader knowledge base that informs clinical practice and research in oncology.

## Case presentation

### Patient information

A 64-year-old male patient, with a prior history of SCC in the same anatomical area (vertex), presented to clinic with a persistent scalp lesion located on the vertex. His medical history was devoid of any significant conditions, including a family history of skin cancer.

### Clinical findings

Upon conducting a thorough clinical examination, there was an elevated, irregular shape scalp ulcer and was found to be immobile. Notably, the patient did not experience tenderness at the site of the lesion. An interesting feature was the mild yellow discoloration that was discernible in the affected area.

### Diagnostic assessment

To establish a comprehensive diagnosis, a series of imaging and diagnostic procedures were initiated. Ultrasound examination unveiled a distinct solid lesion measuring 25x23x13mm, located on the left side of the vertex scalp region. This was accompanied by a constellation of multiple bilateral cervical lymphadenopathies. The most significant lymphadenopathies observed included measurements of 18 mm in the right posterior group, 15.8 mm in right group IV, 26x17mm in the left submandibular region, and 31x20mm extending into the retroclavicular region. Each of these lymph nodes exhibited characteristics highly suggestive of malignancy.

Further radiological assessment through a head and neck CT scan corroborated the presence of the lesion, which was noted as an irregular, well-defined mass measuring 29x22mm. Importantly, this mass did not exhibit any signs of invasion into the surrounding anatomical structures. In addition to this, the scan also highlighted the presence of multiple bilateral cervical lymphadenopathies.

Subsequent to the initial operation, an ultrasound examination uncovered additional pertinent findings. This included the identification of bilateral axillary and inguinal lymph nodes, among which the largest were noted to be 44x24mm in the left axilla and 59x28mm in the left inguinal area. Moreover, a few para-aortic lymph nodes were detected, with the most significant measuring 28x18mm. Notably, the patient’s spleen exhibited enlargement, measuring 12.5 cm.

The diagnostic evaluation was further refined through fine needle aspiration procedures. Notably, a sample obtained from the left cervical lymph node revealed benign lymphoid cells, while conversely, a sample from the left submandibular gland exhibited malignancy, strongly indicative of non-Hodgkin lymphoma.

### Therapeutic intervention

In response to these comprehensive diagnostic findings, a strategic therapeutic plan was formulated. The patient underwent a wide local excision of the scalp lesion. This surgical intervention was followed by a meticulous reconstruction process involving the application of an Orticochea (or pinwheel) flap to cover the excised area. To address the donor site, a graft of new skin was employed.

Simultaneously, both the right and left supraclavicular lymph nodes were surgically excised and subsequently submitted for pathological examination within the same session. Histopathological examination of these specimens unveiled a moderately differentiated grade II invasive SCC, which was associated with lymph node involvement by SCC. Encouragingly, the margins of the scalp lesion were found to be entirely devoid of tumor cells.

In the postoperative phase, a secondary procedure was performed involving the excisional biopsy of the left axillary and left inguinal lymph nodes. These findings reaffirmed the presence of lymphoma, which was conclusively confirmed through immunostaining. Notably, positive immunostaining for CD20, CD10, CD3, and BCL2 provided definitive evidence of the lymphoma diagnosis.

### Follow-up

Following the surgical interventions, the patient was promptly referred to an oncologist to initiate a comprehensive treatment plan. This included a combination of radiotherapy and chemotherapy to effectively manage the underlying malignancies and ensure the best possible outcome for the patient’s health. Rigorous follow-up and monitoring have been instituted to track the patient’s progress and response to therapy.

## Discussion

In this study, a patient with a history of skin cancer had a scalp lesion surgically removed successfully. However, an unexpected lymphoma diagnosis was made during the diagnostic process, leading to treatment with radiotherapy and chemotherapy. This case emphasizes early detection and careful management for skin cancer patients and the importance of comprehensive assessments for concurrent malignancies.

The shared risk factors between SCC and NHL include chronic inflammation and immune dysregulation. Chronic inflammation can persist over long periods of time, causing irreversible organ dysfunction due to tissue damage and/or fibrogenesis [8]. Inflammation has acute and chronic stages, but the link to tumorigenesis is through chronic inflammation. Chronic inflammation is controlled by Tregs and Th2 lymphocytes, which secrete pro-tumorigenic factors, such as IL-4, IL-10, IL-13, and TGF-β6. Immune dysregulation is also a risk factor for both SCC and NHL [9]. Less severe immune dysregulation, in the form of autoimmune conditions and subclinical immune deficiency, has been associated with increased NHL risk [10]. Additionally, genetic predisposition and environmental exposures are also risk factors for both SCC and NHL [11]. Genetic susceptibility studies of lymphoma may serve to identify at risk populations and to elucidate important disease mechanisms. Environmental exposures, such as exposure to radiation, cancer-causing chemicals, or infections, can result in acquired gene changes that increase the risk of developing SCC and NHL [12–14].

Multidisciplinary collaboration plays a critical role in the management of patients with multiple malignancies [15,16]. A team of surgeons, medical oncologists, radiation oncologists, and pathologists worked together to ensure the accurate diagnosis and effective treatment of both SCC and NHL. The implementation of a multidisciplinary approach in cancer patients’ management has been confirmed as beneficial, and it helps clinicians meet the growing needs of cancer patients [17–19]. Multidisciplinary teams can support patient engagement and empowerment, improve treatment efficiency and patient care, and optimize the decision-making process for oncologic patients by improving coordination, communication, and shared decision-making [15]. Multidisciplinary teams will become even more important as cancer management continues to rapidly evolve, and future studies should focus on studies of optimal processes, functions, and success metrics of modern multidisciplinary teams [15].

The treatment plan should be tailored to the individual patient’s needs, taking into account the stage and location of the tumors, the patient’s overall health, and the potential side effects of the treatment. Surgery, radiation therapy, chemotherapy, targeted therapy, and immunotherapy are all potential treatment options for SCC and NHL [20–22]. Neoadjuvant therapy with chemotherapy (chemoradiation) before surgery or after chemotherapy to try to shrink some larger cancers is also an option. In some cases, this might make it possible to use less extensive surgery and remove less tissue [21,22]. The choice of treatment modality depends on the stage and location of the tumors, the patient’s overall health, and the potential side effects of the treatment. A multidisciplinary approach is essential to ensure the best possible outcome for patients with multiple malignancies especially in the head and neck tumors [15,23]. In conclusion, this unique rare case emphasizes the complex interplay of SCC and NHL, necessitating vigilant SCC patient follow-up.

## References

[1] Kallini JR, Hamed N, Khachemoune A. Squamous cell carcinoma of the skin: epidemiology, classification, management, and novel trends. Int J Dermatol. 2015;54(2):130–140. doi:10.1111/ijd.12553. 25428226

[2] Ahmed OF, Kakamad FH, Salih AM, et al. Non-Hodgkin’s Lymphoma presenting as deep venous thrombosis; A case report with literature review. Int J Surg Case Rep. 2020;71:196–198. doi:10.1016/j.ijscr.2020.05.006. 32473551 PMC7260608

[3] Thandra KC, Barsouk A, Saginala K, et al. Epidemiology of Non-Hodgkin’s Lymphoma. Med Sci. 2021;9(1):5. 10.3390/medsci9010005 PMC7930980 33573146

[4] Onajin O, Brewer JD. Skin cancer in patients with chronic lymphocytic leukemia and non-Hodgkin lymphoma. Clin Adv Hematol Oncol. 2012;10(9):571–576. 23073122

[5] Brewer JD, Shanafelt TD, Khezri F, et al. Increased incidence and recurrence rates of nonmelanoma skin cancer in patients with non-Hodgkin lymphoma: a Rochester Epidemiology Project population-based study in Minnesota. J Am Acad. 2015;72(2):302–309. 10.1016/j.jaad.2014.10.028 PMC4508103 25479909

[6] Aso S. Muhialdeen, Jaafar Omer Ahmed, Hiwa O. Baba, et al. Kscien’s List; A New Strategy to Discourage Predatory Journals and Publishers (Second Version). Barw Medical Journal. 2023;1(1):24–26. doi:10.58742/bmj.v1i1.14.

[7] Sørensen HT, Mellemkjaer L, Nielsen GL, et al. Skin cancers and non-hodgkin lymphoma among users of systemic glucocorticoids: a population-based cohort study. J Natl Cancer Inst. 2004;96(9):709–711. doi:10.1093/jnci/djh118. 15126608

[8] Suzuki K. Chronic inflammation as an immunological abnormality and effectiveness of exercise. Biomolecules. 2019;9(6):223. doi:10.3390/biom9060223. 31181700 PMC6628010

[9] Matza Porges S, Shamriz O. Genetics of immune dysregulation and cancer predisposition: two Sides of the Same Coin. Clin Exp Immunol. 2022;210(2):114–127. doi:10.1093/cei/uxac089. 36165533 PMC9750831

[10] Makgoeng SB, Bolanos RS, Jeon CY, et al. Markers of immune activation and inflammation, and non-hodgkin lymphoma: a meta-analysis of prospective studies. JNCI Cancer Spectr. 2018;2(4):pky082. doi:10.1093/jncics/pky082. 30873511 PMC6400235

[11] Robsahm TE, Karagas MR, Rees JR, et al. New malignancies after squamous cell carcinoma and melanomas: a population-based study from Norway. BMC Cancer. 2014;14(1):210. doi:10.1186/1471-2407-14-210. 24645632 PMC3994878

[12] Cerhan JR, Slager SL. Familial predisposition and genetic risk factors for lymphoma. Blood. 2015;126(20):2265–2273. doi:10.1182/blood-2015-04-537498. 26405224 PMC4643002

[13] Moubadder L, McCullough LE, Flowers CR, et al. Linking environmental exposures to molecular pathogenesis in non-hodgkin lymphoma subtypes. Cancer Epidemiol Biomarkers Prev. 2020;29(10):1844–1855. doi:10.1158/1055-9965.EPI-20-0228. 32727723 PMC7541593

[14] Hammood ZD, Salih AM, Latif S, et al. Primary diffuse B-cell non-Hodgkin’s breast lymphoma; A case report with a brief literature review. Annals of Medicine and Surgery. 2022;75 doi:10.1016/j.amsu.2022.103310. PMC8844393 35198180

[15] Selby P, Popescu R, Lawler M, et al. The value and future developments of multidisciplinary team cancer care. Am Soc Clin Oncol Educ Book. 2019;39:332–340. ():doi:10.1200/EDBK_236857. 31099640

[16] Taberna M, Gil Moncayo F, Jané-Salas E, et al. The Multidisciplinary Team (MDT) approach and quality of care. Front Oncol. 2020;10:85. doi:10.3389/fonc.2020.00085. 32266126 PMC7100151

[17] Shore ND, Morgans AK, El-Haddad G, et al. Addressing challenges and controversies in the management of prostate cancer with multidisciplinary teams. Target Oncol. 2022;17(6):709–725. doi:10.1007/s11523-022-00925-7. 36399218 PMC9672595

[18] Saini KS, Taylor C, Ramirez A-J, et al. Role of the multidisciplinary team in breast cancer management: results from a large international survey involving 39 countries. Ann Oncol. 2012;23(4):853–859. doi:10.1093/annonc/mdr352. 21821551

[19] Petrella F, Radice D, Guarize J, et al. The impact of multidisciplinary team meetings on patient management in oncologic thoracic surgery: a single-center experience. Cancers (Basel). 2021;13(2):228. doi:10.3390/cancers13020228. 33435181 PMC7827504

[20] Ansell SM, Armitage J. Non-Hodgkin lymphoma: diagnosis and treatment. Mayo Clin Proc. 2005;80(8):1087–1097. doi:10.4065/80.8.1087. 16092591

[21] Adult Hodgkin Lymphoma Treatment (PDQ®): Patient Version. In Bethesda (MD). 2002.

[22] Kim JYS, Kozlow JH, Mittal B, et al. Guidelines of care for the management of cutaneous squamous cell carcinoma. J Am Acad Dermatol. 2018;78(3):560–578. doi:10.1016/j.jaad.2017.10.007. 29331386 PMC6652228

[23] Aso S. Muhialdeen, Hiwa O. Baba, Abdulwahid M. Salih, et al. Redo thyroidectomy modified technique to eliminate complications: a cohort study. Barw Medical Journal.2023;1(2):7–11. doi:10.58742/bmj.v1i2.27.

